# Ultrasmall Fe_2_O_3_ nanoparticles/MoS_2_ nanosheets composite as high-performance anode material for lithium ion batteries

**DOI:** 10.1038/srep42772

**Published:** 2017-02-20

**Authors:** Bin Qu, Yue Sun, Lianlian Liu, Chunyan Li, Changjian Yu, Xitian Zhang, Yujin Chen

**Affiliations:** 1 Key Laboratory of In-Fiber Integrated Optics, Ministry of Education and College of Science, Harbin Engineering University, Harbin 150001, China; 2 Key Laboratory for Photonic and Electronic Bandgap Materials, Ministry of Education and School of Physics and Electronic Engineering, Harbin Normal University, Harbin 150025, China

## Abstract

Coupling ultrasmall Fe_2_O_3_ particles (~4.0 nm) with the MoS_2_ nanosheets is achieved by a facile method for high-performance anode material for Li-ion battery. MoS_2_ nanosheets in the composite can serve as scaffolds, efficiently buffering the large volume change of Fe_2_O_3_ during charge/discharge process, whereas the ultrasmall Fe_2_O_3_ nanoparticles mainly provide the specific capacity. Due to bigger surface area and larger pore volume as well as strong coupling between Fe_2_O_3_ particles and MoS_2_ nanosheets, the composite exhibits superior electrochemical properties to MoS_2_, Fe_2_O_3_ and the physical mixture Fe_2_O_3_+MoS_2_. Typically, after 140 cycles the reversible capacity of the composite does not decay, but increases from 829 mA h g^−1^ to 864 mA h g^−1^ at a high current density of 2 A g^−1^. Thus, the present facile strategy could open a way for development of cost-efficient anode material with high-performance for large-scale energy conversion and storage systems.

Owing to their high energy densities and environmental benignity, lithium ion batteries (LIBs) have been used as potential power sources for various electronic devices and equipments, ranging from a tiny music player to a massive sports car^1^,^2^. However, the commercial graphite anode of LIBs is difficult to satisfy the requirements of high power equipment of the modern society due to its low specific capacity (372 mAh g^−1^). Thus, alternative anode materials with good electrochemical performances are particularly desirable. 2H-MoS_2_, as a typical member of transition metal dichalcogenides, is composed of a layer of molybdenum atoms sandwiched between two layers of sulphur atoms. The spacing between neighboring layers is 0.615 nm, significantly larger than that of graphite (0.335 nm), and the weak van der Waals forces between the layers allows Li ions to diffuse without a significant increase in volume, leading to high-performance of MoS_2_ as anode material than that of graphite^3^. The theoretical capacity of MoS_2_ is as high as 670 mAh g^−1^, resulting from a conversion reaction of MoS_2_+4Li^+^ + 4e^−^ → Mo+2Li_2_S^4^,^5^. Furthermore, MoS_2_ surface exists many unsaturated sulfur dangling bonds, which will also be involved in the charge and discharge reactions^6^, and consequently the actual capacity of MoS_2_ is often higher than the theoretical value^7^,^8^,^9^. Recently, in order to improve the reversible capacity of MoS_2_ many strategies were developed to synthesize various MoS_2_ nanostructures including exfoliated MoS_2_^10^ and hollow MoS_2_ nanosheet assemblies, nanotubes^11^,^12^, nanoboxes^13^,^14^, MoS_2_@void@MoS_2_^15^, and hollow nanospheres^16^,^17^. Unfortunately, due to the poor conductivity, the MoS_2_ materials exhibited inferior cycling stability and rate performance, which impedes their practical application^10^. One efficient solution is to introduce carbon materials, such as graphene nanosheets^18^,^19^,^20^,^21^,^22^,^23^,^24^,^25^,^26^,^27^,^28^,^29^, carbon nanotubes^30^,^31^,^32^, carbon nanospheres^33^, carbon fiber cloth^34^,^35^, mesoporous carbon^36^ to improve the electrical conductivity of the composite materials. However, due to the lower capacity of these carbon materials, the overall energy density of the composite material would be suppressed.

Because of its high theoretical capacity (1005 mA h g^−1^), low cost, abundance in nature, and environmental benignity Fe_2_O_3_ is another promising anode material^37^,^38^. Especially when Fe_2_O_3_ in ultrasmall size (5∼10 nm) can exhibit high rate electrochemical performances^37^,^38^. Firstly, the ultrasmall size can greatly mitigate the volume expansion/contraction of Fe_2_O_3_ particles during charge/discharge. Secondly, a high lithium ion flux can be achieved by the large surface area provided by the ultrasmall particles^39^. More importantly, due to the extremely short distance for lithium ions transportation within ultrasmall particles, the rate capability of lithium insertion/removal can be significantly enhanced^40^. However, the nanostructured Fe_2_O_3_ exhibited a poor cycling stability due to structural damage during charging/discharging process^37^.

Herein, we report a facile method to grow ultrasmall Fe_2_O_3_ nanoparticles on 2H-MoS_2_ nanosheets, where MoS_2_ nanosheets in the composite can serve as scaffolds, efficiently buffering the large volume changes of Fe_2_O_3_ during charging/discharging process, whereas the ultrasmall Fe_2_O_3_ nanoparticles mainly provide the specific capacity of the anode as well as the enhanced electrical conductivity. Furthermore, strong coupling between Fe_2_O_3_ and MoS_2_ nanosheets, elucidated by X-ray photoelectron spectrum measurements, facilitates a rapid charge transfer. In addition, MoS_2_ nanosheets in the composite can also contribute to the total capacity of the anode. As a consequence, the composite prepared here exhibited superior electrochemical performance for anode material for Li-ion battery.

## Results

**Figure 1:**
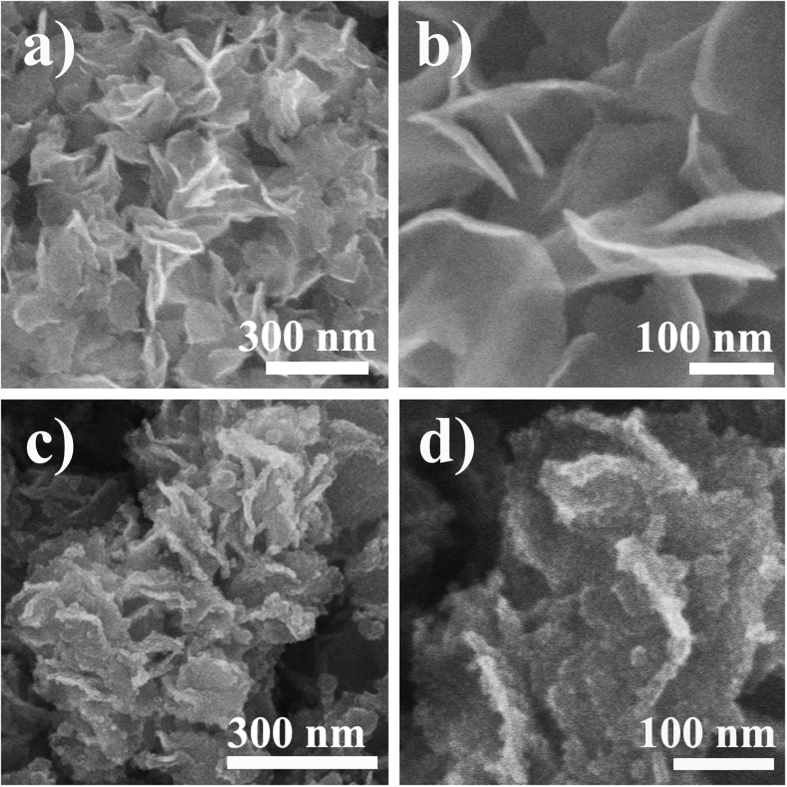
Structrual characterization of MoS_2_ nanosheets and Fe_2_O_3_/MoS_2_ composite. (**a**,**b**) SEM of MoS_2_ nanosheets, and (**c**,**d**) SEM of Fe_2_O_3_/MoS_2_ composite.

**Figure 2:**
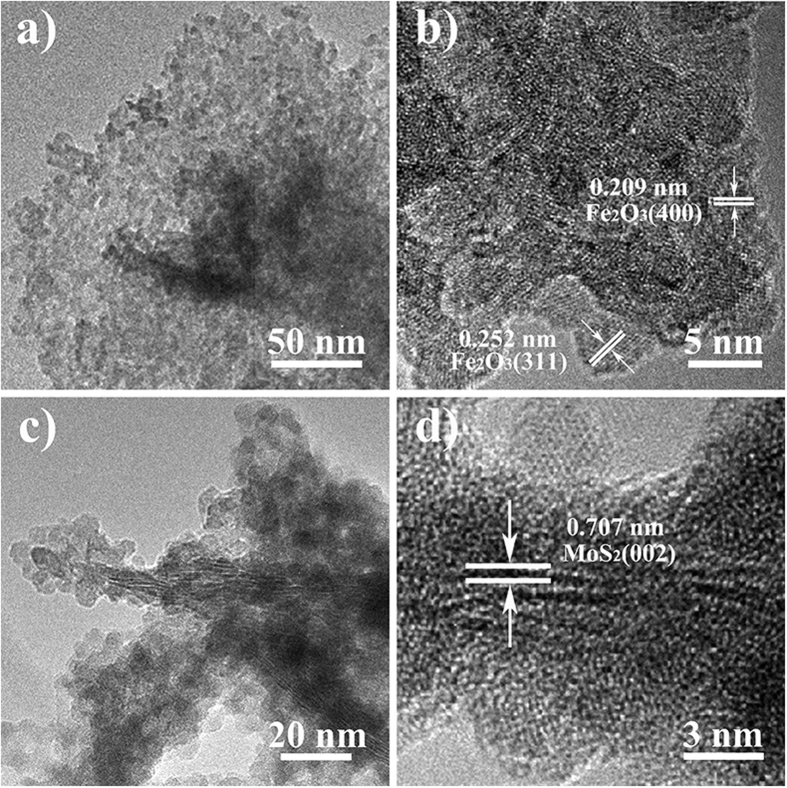
TEM image of Fe_2_O_3_/MoS_2_ composite. (**a**,**b**) TEM and HRTEM images taken from basal plane, and (**c**,**d**) cross-section TEM and HRTEM image of basal plane.

SEM images (Fig. 1a and b) show that the as-prepared MoS_2_ exhibits sheet-like morphology with a thickness and a lateral length of about 10 and 400 nm, respectively, similar to that reported previously^41^. After Fe_2_O_3_ coating, the MoS_2_ exhibits a similar morphology and lateral length to that of the pristine MoS_2_ nanosheets, but the surface becomes drastically rough (Fig. 1c), in sharp contrast to the smooth surface of the pristine MoS_2_ nanosheets (Fig. 1b). From the high-magnification SEM image of the composite (Fig. 1d)), it can also be found that many ultrasmall particles are anchored on both sides of basal planes of MoS_2_ nanosheets. Figure 2a shows a TEM image taken from the basal plane of MoS_2_ nanosheets in the composite. It can be found that Fe_2_O_3_ nanoparticles are uniformly and densely deposited on the surface of the MoS_2_ nanosheets. The size distribution plot (Figure S1) indicates that the average size of Fe_2_O_3_ nanoparticles is about 4.0 nm. Most lattice fringes of the Fe_2_O_3_ nanoparticles in the high-resolution TEM (HRTEM) image (Fig. 2b) are not resolved well, revealing the weak degree of crystallizations of the Fe_2_O_3_ nanoparticles. The labeled lattice spacing for Fe_2_O_3_ nanoparticles in the HRTEM image is about 0.209 nm and 0.252 nm, which can be assigned to the (400) plane and (311) plane of Fe_2_O_3_, respectively. The fast Fourier transformation (FFT) technique confirms the crystal nature of Fe_2_O_3_ on the MoS_2_ nanosheets (Figure S2). Cross-section TEM image (Fig. 2c) reveals that the utrasmall Fe_2_O_3_ nanoparticles mainly disperse on the basal planes of the MoS_2_ nanosheets, in which the lattice fringes corresponding to (002) plane can be clearly observed. HRTEM image (Fig. 2d) reveals that the interlayer distance of the (002) plane of the MoS_2_ nanosheets is about 0.707 nm, larger than the value (0.615 nm) of bulk MoS_2_.

**Figure 3:**
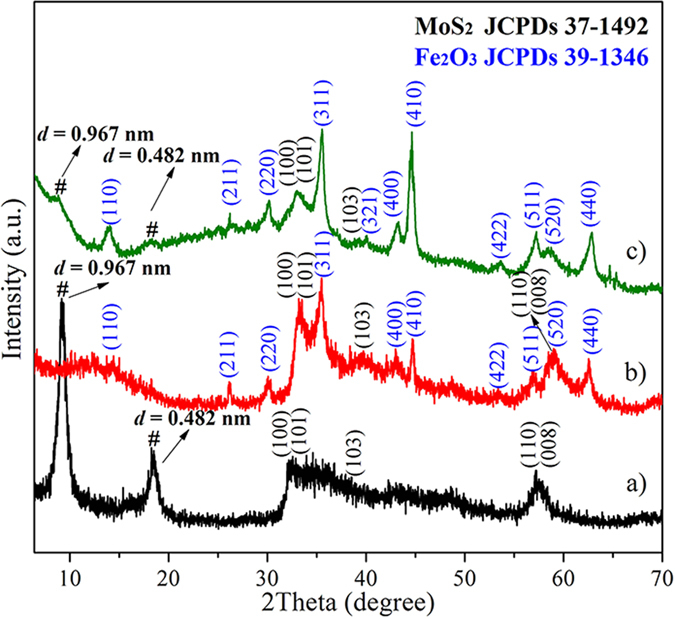
XRD patterns. (**a**) MoS_2_ nanosheets, (**b**) Fe_2_O_3_/MoS_2_ composite and (**c**) the physical mixture Fe_2_O_3_+MoS_2_.

The crystal structures of the samples were examined using X-ray diffraction (XRD) measurement. Figure 3a shows the XRD pattern of the pristine MoS_2_ nanosheets, in which the peaks located at 2*θ* = 32.2° corresponds to the (100) and (101) planes, 2*θ* = 38.3° corresponds to the (103) plane and the peaks located at 2*θ* = 57.3° corresponds to the (110) and (008) planes of 2H-MoS_2_ (JCPDS No. 37–1492). Compared to 2H-MoS_2_ bulk, these peaks slightly shift toward low-angle region, revealing the slightly enlarged lattice distances along the basal planes of 2H-MoS_2_. Similar to the previous report^41^, two additional peaks located at 9.2° and 18.5°, marked by “#”, are also observed at low-angle region. The corresponding *d*-spacings calculated according to the Bragg equation are 0.96 and 0.48 nm, respectively. The diploid relation between the *d*-spacings reveals that the MoS_2_ nanosheets possess a new lamellar structure with a larger interlayer spacing of 0.96 nm than that of 0.615 nm in bulk 2H-MoS_2_^34^,^41^,^42^. The enlarged interlayer spacing may be related to the synthesis conditions^28^,^34^,^41^,^42^. As previously reported^41^, when the temperature was lower than 180 °C, the MoS_2_ nanosheets with enlarged interlayer spacing (0.95 nm) could be obtained in alkaline media; while the temperature was increased to 220 °C, the interlayer distance of the nanosheets kept the same value as that in bulk MoS_2_^41^. On the other hand, the enlarged interlayer spacing could be achieved in the media containing urea at 220 °C; however, when the pH in the media was decreased by replacing urea with ammonium fluoride, even at the same temperature the phenomenon did not occur^42^. Therefore, the alkalinity and the synthetic temperature seriously affect the interlayer distance of the MoS_2_ nanosheets. Under the experimental conditions such as strong alkaline media and low temperature, oxygen species may incorporate more easily with MoS_2_, leading to the different lamellar structure with a enlarged interlayer spacing than that of 0.615 in bulk 2H-MoS_2_^34^,^41^,^42^. However, the new lamellar structure is thermodynamically unstable. After annealing the MoS_2_ nanosheets at 500 °C for 3 h under an Ar flow, XRD analysis was carried out. As shown in Figure S3, the diffraction peaks at 9.2° and 18.5° disappear, while all the resolved peaks can be assigned to thermodynamically stable 2H-MoS_2_ (JCPDS No. 37–1492). After Fe_2_O_3_ coating, the peak corresponding to the (002) plane is suppressed significantly, further suggesting that uniform and dense nanoparticles are deposited on the both sides of the basal plane of the MoS_2_ nanosheets (Fig. 3b). In addition, the peaks from the Fe_2_O_3_ and MoS_2_ can also be identified in the XRD pattern. The diffraction peaks at 2*θ* = 33.4°can be assigned to (100) and (101) planes, 2*θ* = 39.5°can be assigned to (103) plane and 2*θ* = 58.5° can be assigned to (110) and (008) planes of 2H-MoS_2_, respectively, whereas those at 2*θ* = 14.5°, 26.1°, 30.2°, 35.5°, 43.2°, 44.6°, 53.6°, 57.1°, 59.6° and 62.7° can be indexed to (110), (211), (220), (311), (400), (410), (422), (511), (520)and (440) planes of Fe_2_O_3_ (JCPDS no. 39–1346), respectively. Notably, the peak position of (110) plane in Fig. 3a is different from that in Fig. 3b. This is because the pristine MoS_2_ nanosheets with the enlarged interlayer spacing are thermodynamically unstable phases, while the MoS_2_ nanosheets in Fe_2_O_3_/MoS_2_ composite, which were annealing at 500 °C for 3 h under an Ar flow, are thermodynamically stable. They had different lamellar structures, leading to significantly difference in XRD results (Fig. 3 and Figure S4). Compared to the XRD pattern of the annealed MoS_2_ nanosheets with that of the Fe_2_O_3_/MoS_2_ composite, the peak position of (110) plane is almost identical (Figure S5). The above results demonstrate that crystalline Fe_2_O_3_ nanoparticles are successfully anchored on the surface of MoS_2_ nanosheets. Figure 3c) shows the XRD pattern of the physical mixture Fe_2_O_3_ + MoS_2_, in which the diffraction peaks from both Fe_2_O_3_ and MoS_2_ can be observed. Notably, the diffraction peak corresponding to expanded (002) plane of MoS_2_ nanosheets is still visible, significantly different from that of the Fe_2_O_3_/MoS_2_ composite.

**Figure 4:**
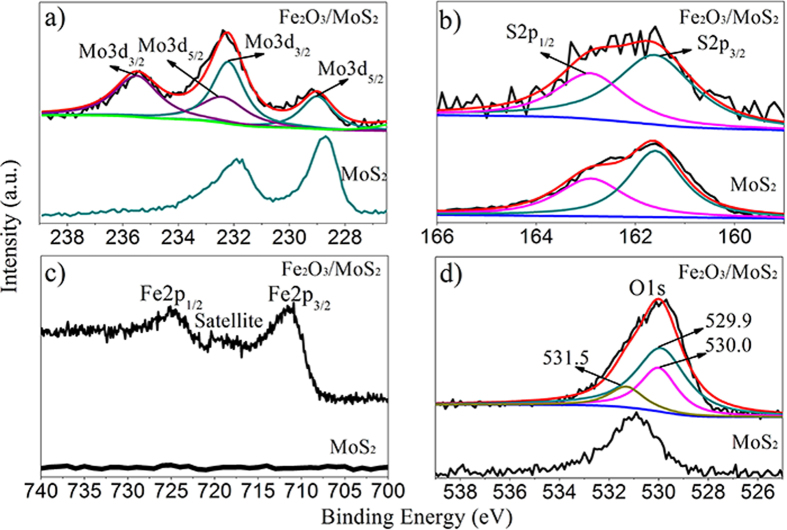
XPS spectra of Fe_2_O_3_/MoS_2_ composite and MoS_2_ nanosheets. (**a**) Mo 3d XPS spectrum, (**b**) S 2p XPS spectra, (**c**) Fe 2 P XPS spectrum, and (**d**) O 1 s XPS spectrum.

X-ray photoelectron spectroscopy (XPS) analysis was carried out to determine surface chemical compositions and valence states of the Fe_2_O_3_/MoS_2_ composite and the MoS_2_ nanosheets. Figure 4a shows the high-resolution XPS spectra of Mo 3d core level for the two samples. Two peaks at 231.9 eV and 228.7 eV are observed in the Mo 3d spectrum of the MoS_2_ nanosheets, corresponding to Mo^4+^ species. After coating Fe_2_O_3_, the two peaks shift to high binding energy side by an approximately 0.3 eV. The shift of the binding energy indicates that electron transfer from MoS_2_ to Fe_2_O_3_ occurs. It can be concluded that the strong coupling between MoS_2_ and Fe_2_O_3_ is presented in the composite. However, such shift in the physical mixture MoS_2_+Fe_2_O_3_ does not occur (Figure S6), revealing the advantage of our method for preparation of the Fe_2_O_3_/MoS_2_ composite. In addition, two additional peaks at 235.6 eV and 232.5 eV are assigned to Mo^6+^ species^43^,^44^,^45^, suggesting the surface oxidization of MoS_2_ due to the electron transfer. In the XPS spectrum of S 2p core level for the pure MoS_2_ nanosheets, the main doublet located at binding energies of 161.6 and 162.9 eV correspond to the S 2p_3/2_ and S 2p_1/2_, respectively (Fig. 4b)^41^. There are no obvious shift of the binding energy of the two peaks for the Fe_2_O_3_/MoS_2_ composite, implying that the Fe_2_O_3_ coating has little effect on the valence states of the S species. In the Fe 2p core level spectrum for the Fe_2_O_3_/MoS_2_ composite, the peaks at 711.3 eV, 719.2 eV and 724.8 eV represent the binding energies of Fe 2p_3/2_, shake-up satellite Fe 2p_3/2_, and Fe 2p_1/2_ of Fe^3+^ species, respectively (Fig. 4c)^46^,^47^. These values are consistent with the data of Fe_2_O_3_ reported by the previous literatures^48^,^49^, confirming the existence of Fe_2_O_3_ in the Fe_2_O_3_/MoS_2_ composite. Compared to the XPS spectra of Fe_2_O_3_ in the physical mixture MoS_2_+Fe_2_O_3_, the peaks for Fe 2p_1/2_ and Fe 2p_3/2_ shift to low binding energy side, further confirming the coupling effect between Fe_2_O_3_ and MoS_2_ in the Fe_2_O_3_/MoS_2_ composite. The peaks 529.9 and 530.0 eV in the high-resolution XPS spectrum of O 1 s core level for the Fe_2_O_3_/MoS_2_ composite can be assigned to oxygen in the lattice (Fe−O)^50^,^51^ and oxygen in the lattice (Mo−O)^41^, respectively (Fig. 4d). Besides, the peak at 531.5 eV is associated to the hydroxyl oxygen.

**Figure 5:**
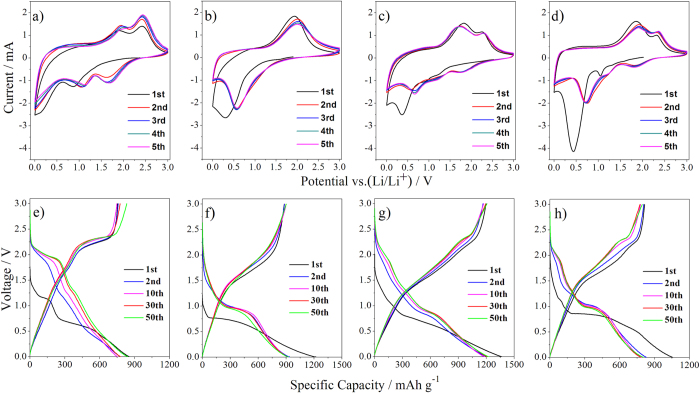
CV curves of (**a**) MoS_2_ nanosheets, (**b**) Fe_2_O_3_, (**c**) Fe_2_O_3_/MoS_2_ composite and (**d**) the physical mixture Fe_2_O_3_+MoS_2_, and charging–discharging curves of (**e**) MoS_2_ nanosheets, (**f**) Fe_2_O_3_, (**g**) Fe_2_O_3_/MoS_2_ composite and (**h**) the physical mixture Fe_2_O_3_+MoS_2_.

Cyclic voltammogram (CV) tests for coin cells of the pristine MoS_2_ nanosheets were recorded at ambient temperature in the voltage range of 0.01–3 V at a scan rate of 1 mV s^−1^ for the initial five cycles, as shown in Fig. 5a. The peak of 0.87 V in the first cathodic scanning is ascribed to the intercalation of lithium ion on different defect sites in MoS_2_ to form Li_x_MoS_2_. In the following cathodic scanning, two new reduction peaks at approximately 1.65 V and 1.15 V are observed, which are due to the conversion of S to Li_2_S and the association of Li with Mo respectively^52^,^53^. During the anodic scans, two peaks at 1.92 and 2.42 V are clearly observed and maintain for the subsequent sweeps, which are related to the conversion reaction of Mo and Li_2_S to MoS_2_ phase^54^,^55^. As for the pure Fe_2_O_3_ in Fig. 5b, the reduction peaks at 0.36 V is observed in the first cycle, and its position shifts to 0.57 V at the following scanning, which is attributed to the reduction of Fe(III) to Fe(0). In the anodic scans, the oxidation peak at 1.95 V is the oxidation of Fe to Fe_2_O_3_. As for the Fe_2_O_3_/MoS_2_ composite, three reduction peaks at 0.36 V, 0.87 V and 1.20 V are observed in the first cycle (Fig. 5c). The reduction peaks locate at 0.36 V and 0.87 V during the first anodic scan can match anodic scan peaks of pure Fe_2_O_3_ and pure MoS_2_, respectively. The peak located at 1.20 V shows a same start shoulder at ~1.05 V with pure MoS_2_, which suggest the same lithiation process of MoS_2_^8^. At the following scanning, however, these peaks shift to 0.65 V, 1.15 V and 1.75 V respectively. The peak at 1.15 V is related to the conversion of MoS_2_ to Mo and Li_2_S, while two other peaks at 1.75 and 0.65 V are attributed to the formation of Li_x_Fe_2_O_3_ due to the lithiation of Fe_2_O_3_ and the reduction of Fe(III) to Fe(0), respectively^56^,^57^,^58^,^59^,^60^. In the anodic scans, the oxidation peaks at 1.82 V and 2.40 V stand for oxidation of Mo to MoS_2_ and Fe to Fe_2_O_3_, respectively. The CV results demonstrate that both Fe_2_O_3_ and MoS_2_ in the composite contribute to the capacity of the composite. As for the physical mixture Fe_2_O_3_+MoS_2_ (Fig. 5d), three reduction peaks at 0.43 V, 1.04 V and 1.30 V are found in the first cycle, and then their positions shift to 0.71 V, 1.16 V and 1.81 V after the following scanning, respectively, which are close to the positions of the corresponding peaks for Fe_2_O_3_/MoS_2_ composite (Fig. 5c). In the anodic scans, the oxidation peaks are also in accordance with Fe_2_O_3_/MoS_2_ composite. The observations suggest that similar electrochemical reactions occur for Fe_2_O_3_+MoS_2_ and Fe_2_O_3_/MoS_2_ composites during charging/discharging process. However, the physical mixture Fe_2_O_3_+MoS_2_ has larger irreversible capacity than the Fe_2_O_3_/MoS_2_ composite, as shown in Fig. 5c and d, suggesting advantage of coupling ultrasmall Fe_2_O_3_ nanoparticles with MoS_2_ nanosheets. Figure 5e–h show the voltage-capacity curves of pristine MoS_2_ nanosheets, pure Fe_2_O_3_, Fe_2_O_3_/MoS_2_ composite and physical mixture Fe_2_O_3_+MoS_2_ at a current density of 100 mA g^−1^. The initial discharging/charging capacities of the Fe_2_O_3_/MoS_2_ composite are 1366/1207 mA h g^−1^, greatly larger than that of pure MoS_2_ nanosheets (854/754 mAh g^−1^), pure Fe_2_O_3_ (1218/879 mAh g^−1^) and the physical mixture Fe_2_O_3_+MoS_2_ (1056/815 mAh g^−1^). Furthermore, the Fe_2_O_3_/MoS_2_ composite has a Coulombic efficiency of 88.4% at the first cycle, much higher than that of the physical mixture Fe_2_O_3_+MoS_2_ (77.2%), consistent with the CV results. The energy efficiency (discharge energy/charge energy) of the Fe_2_O_3_/MoS_2_ composite are 43%~58% (Figure S7). The larger specific capacity and higher Coulombic efficiency of the Fe_2_O_3_/MoS_2_ composite indicate that coupling ultrasmall Fe_2_O_3_ particles with MoS_2_ nanosheets is an efficient strategy to improve the electrochemical performance of the MoS_2_ nanosheets.

**Figure 6:**
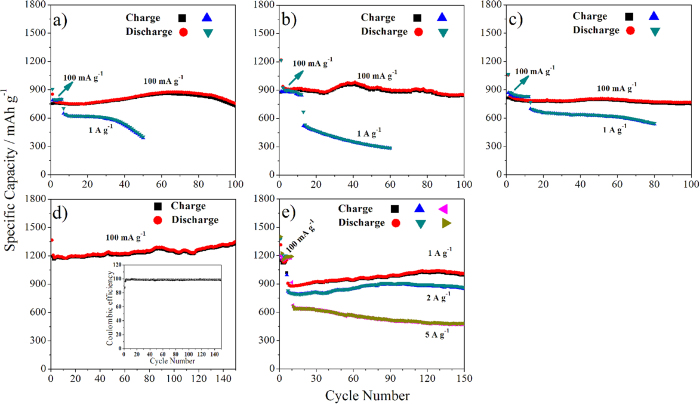
Cycling stability of samples at different current densities. (**a**) MoS_2_ nanosheets at 100 and 1000 mA g^−1^, (**b**) Fe_2_O_3_ at 100 and 1000 mA g^−1^, (**c**) the physical mixture Fe_2_O_3_+MoS_2_ at 100 and 1000 mA g^−1^, (**d**) Fe_2_O_3_/MoS_2_ composite at 100 mh g^−1^, the inset showing the corresponding Coulombic efficiency and (**e**) Fe_2_O_3_/MoS_2_ composite at 1000 mA g^−1^, 2 000 mA g^−1^ and 5000 mA g^−1^ (initial 6 cycles at 100 mA g^−1^).

To confirm the superiority of the Fe_2_O_3_/MoS_2_ composite as an anode material over the pristine MoS_2_ nanosheets and the physical mixture Fe_2_O_3_+MoS_2_ in the lithium storage performance, we compared their cycling behaviors at different current densities (Fig. 6). Clearly, MoS_2_ nanosheets deliver an initial capacity of 854 mAhg^−1^ at a current density of 100 mA g^−1^ (Fig. 6a), higher than its theoretical value due to its ultra-thin nanosheets for lithium storage. However, obvious capacity decay is witnessed when cycled at a high current density (Fig. 6a). For example, the capacity decreases from 638 mA h g^−1^to 449 mA h g^−1^ at 1 A g^−1^ only after 40 cycles. This phenomenon is probably due to the exfoliation of the ultra-thin MoS_2_ nanosheets during discharging/charging process. Similarly, the pure Fe_2_O_3_ delivers an initial discharge capacity of 1219 mAh g^−1^at a current densities of 100 mA g^−1^, and the capacity decreases from 533 mAh g^−1^ to 287 mAh g^−1^ at 1 A g^−1^ only after 55 cycles (Fig. 6b). As the MoS_2_ nanosheets are mixed with Fe_2_O_3_ mechanically, slightly better cycling stability at the current densities of 100 mA g^−1^ and 1 A g^−1^ than the pristine MoS_2_ nanosheets can be achieved, as shown in Fig. 6c. However, the physical mixture still shows poor cycling stability at a high current density. For example, the discharge capacity of the physical mixture decreases from 698 mA h g^−1^ to 545 mA h g^−1^ after 80 cycles at a high current density of 1 A g^−1^ (Fig. 6c). In contrast, the Fe_2_O_3_/MoS_2_ composite exhibits drastically enhanced rate capability (Fig. 6d,e and Figure S8). Furthermore, the Fe_2_O_3_/MoS_2_ composite shows an excellent cycling durability at different current densities. For example, the Fe_2_O_3_/MoS_2_ composite delivers an initial discharge capacity of 1366 mAh g^−1^ at current densities of 100 mA g^−1^. The capacity does not decay after 150 cycles, but gradually increases to 1350 mA h g^−1^ with a high Coulombic efficiency of >98.7% (Fig. 6d). Surprisingly, even at high current densities of 1 and 2 A g^−1^, the composite also exhibits excellent cycling stability (Fig. 6e). The capacity of the Fe_2_O_3_/MoS_2_ composite increases from 908 mA h g^−1^ to 1011 mA h g^−1^ at 1A g^−1^, and from 829 mA h g^−1^ to 864 mA h g^−1^ at 2 A g^−1^ after 140 cycles (Fig. 6e). The cycling performance is inferior to that of MoS_2_/graphene composite with the capacity of 907 mA h g^−1^ after 400 cycles^29^, however, comparable or superior to most other Fe_2_O_3_ and MoS_2_ or their composites, which is summarized in Table S1^8^,^13^,^21^,^37^,^61^,^62^,^63^,^64^,^65^,^66^,^67^,^68^,^69^,^70^,^71^. For example, the capacity of Fe_2_O_3_ nanoparticles was only about 300 mAh g^−1^ at ca. 100 m A g^−1^ after 100 cycles^37^; the capacity of CNTs–MoS_2_ was 737 mAh g^−1^ at 100 m A g^−1^ after 30 cycles^69^. Furthermore, when the current density is increased to 5 A g^−1^, the composite shows a relatively bad cycling durability, but still delivers a capacity of 481 mAh g^−1^ after 140 cycles. To reveal the charge/discharge stability of anode, the SEM and elemental mapping analyses of Fe_2_O_3_/MoS_2_ composite after 100 cycles were carried out. After the cycling, the utrasmall Fe_2_O_3_ nanoparticles are still resolved in the basal planes of the MoS_2_ nanosheets, as shown in Figure S9. Elemental mapping images (Figure S10) reveal that Fe and Mo elements are uniformly distributed in the composite. These results above demonstrate that the Fe_2_O_3_/MoS_2_ composite exhibit significantly enhanced capacity, rated capability and cycling stability compared to the pristine MoS_2_ nanosheets, the pure Fe_2_O_3_ and the physical mixture Fe_2_O_3_+MoS_2_. Notably, the electrochemical performance of Fe_2_O_3_/MoS_2_ composite was measured at room temperature. It is well known that the electrochemical performance of the anode material for LIBs is suppressed significantly at the ambient temperature lower than 0 °C. However, as previously reported, the specific capacity of MoS_2_/G electrode at −20 °C still remained ca. 700 mAh g^−1^ at 100 mA g^−1^^29^. This result indicates that MoS_2_-based anode material may be used at low-temperature environment. The electrochemical performance of our Fe_2_O_3_/MoS_2_ composite at such low-temperature environment is studied under way.

## Discussion

**Figure 7:**
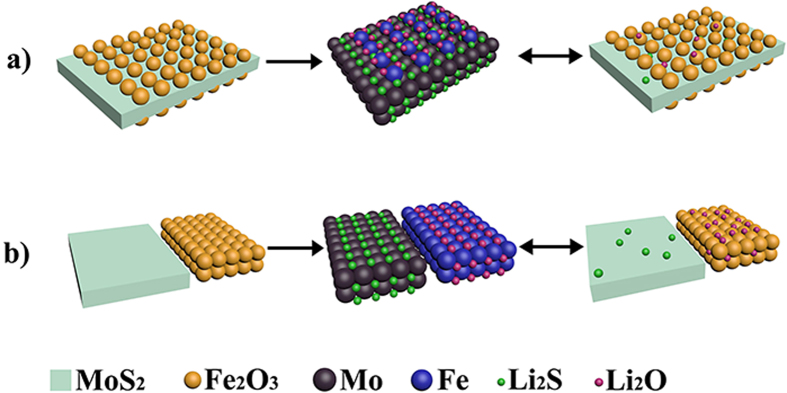
(**a**) Schematic illustration of the irreversible Li_2_O converse to Li^+^ for the Fe_2_O_3_/MoS_2_ composite. Due to high electrochemical activity of Mo metal clusters and efficient contact between Mo metal clusters and the irreversible Li_2_O, the irreversible Li_2_O can converse to Li^+^ after the charging process, and (**b**) Schematic illustration of the irreversible Li_2_O converse to Li^+^ for the physical mixture Fe_2_O_3_+MoS_2_. The conversion of the irreversible Li_2_O to Li^+^ will be greatly suppressed because the efficient contact between Mo and Li_2_O is more difficultly available.

The excellent electrochemical properties of the Fe_2_O_3_/MoS_2_ composite, as evidenced by a remarkably increased reversible capacity, improved rate capability, and robust long-term stability even at a high current density, indicates that the Fe_2_O_3_/MoS_2_ composite is favorable for superior anode materials for Li-ion battery. The following factors can be attributed to the improved electrochemical properties of the Fe_2_O_3_/MoS_2_ composite. First, unlike some designed composites^21^,^61^,^63^,^68^,^69^,^71^, both Fe_2_O_3_ and MoS_2_ in our composite can contribute to the total capacity of the anode, elucidated by CV measurements (Fig. 5c). The high reversible capacity of the Fe_2_O_3_/MoS_2_ composite may also be related to its unique heteronanostructure character. As we know, during the first discharge process, Fe_2_O_3_ reacts with Li^+^, and then Fe and Li_2_O will gradually produce (Equation 1)^72^; however, only partial Li_2_O can reversibly converse to Li^+^ during the subsequent charging process, leading to a high irreversible capacity of Fe_2_O_3_-based anodes. On the other hand, during the first discharging process of MoS_2_, amorphous Mo metal clusters will form (Equation 2) and disperse on the surface of MoS_2_. The Mo metal clusters have highly electrochemical activity^73^,^74^. Considering the unique heteronanostructure of the Fe_2_O_3_/MoS_2_ composite, the Mo metal clusters on the MoS_2_ surface can efficiently contact with Li_2_O and make the irreversible Li_2_O converse to Li^+^, as shown in Fig. 7a. As a result, the Fe_2_O_3_/MoS_2_ composite shows a low irreversible capacity and a high Coulombic efficiency of 88.4% at the first cycle. As for the physical mixture Fe_2_O_3_+MoS_2_, since the efficient contact between Mo and Li_2_O is more difficultly available, the conversion of Li_2_O to Li^+^ will be greatly suppressed (Fig. 7b). Consequently, the physical mixture Fe_2_O_3_+MoS_2_ shows a high irreversible capacity and a low Coulombic efficiency at the first cycle (Fig. 5d and h).


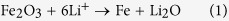



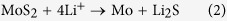


**Figure 8:**
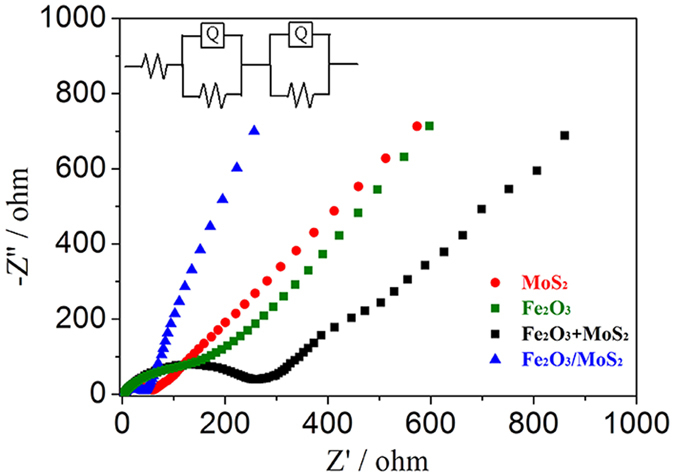
Nyquist plots for samples from 100 kHz to 0.01 Hz.

Second, the average Fe_2_O_3_ size is approximately 4.0 nm, which can shorten the Li ion transfer length and then facilitate the improvement of the rate capability. Additionally, nitrogen adsorption–desorption isotherms show that Brunauer–Emmett–Teller (BET) surface areas and cumulative volume of pores were 12.5 m^2^ g^−1^ and 0.06 cm^3^ g^−1^ for the pristine nanosheets, around two times lower than those of the composite (23.1 m^2^ g^−1^ and 0.12 cm^3^ g^−1^), as shown in Figure S11 and Figure S12. The bigger BET surface area and larger pore volume not only allow for fast Li-ion diffusion, but also buffer the volume changes accompanying the Li charging and discharging processes^28^. Third, the strong coupled interfaces boost a rapid interfacial charge transfer, leading to excellent rate capability of the Fe_2_O_3_/MoS_2_ composite, as evidenced by electrochemical impedance measurements. Nyquist plots (Fig. 8) shows that the Fe_2_O_3_/MoS_2_ composite has a charge transfer resistance (*R*_ct_) of 39.9 Ω, greatly smaller than that of the physical mixture Fe_2_O_3_+MoS_2_ (238.9 Ω), the pure Fe_2_O_3_ (136.0 Ω) and the pristine MoS_2_nanosheets (51.3 Ω), at high frequency 100000 Hz, Low frequency 0.01 Hz and amplitude 0.005 V. The strong coupling between MoS_2_ and Fe_2_O_3_ implies that small Fe_2_O_3_ nanoparticles are tightly anchored on the MoS_2_ scaffolds, facilitating long-term stability of the Fe_2_O_3_/MoS_2_ composite even at a high current density. Taken together, the synergistic effect of two excellent anode materials and the unique structural features of the composite make it an attractive candidate for anode material for Li-ion battery.

In summary, a facile and cost-effective strategy was developed to anchor ultrasmall Fe_2_O_3_ nanoparticles on the surface of MoS_2_ nanosheets. Due to the synergistic effect of two excellent anode materials and the unique structural feature, the Fe_2_O_3_/MoS_2_ composite exhibits excellent electrochemical properties, including a remarkably increased reversible capacity, improved rate capability, and long-term stability even at a high current density. After 140 cycles the reversible capacity of the composite does not decay, but increases from 829 mA h g^−1^ to 864 mA h g^−1^ at a high current density of 2 A g^−1^, outperforming other MoS_2_- and iron oxide-based anode materials previously reported. Thus, the facile strategy may open a way for development of cost-efficient anode material with high-performance for large-scale energy conversion and storage systems.

## Methods

### Synthesis of samples

MoS_2_ nanosheets were first synthesized by a solution-based method^40^. Simply, (NH_4_)_6_Mo_7_O_24_·4H_2_O (1 mmol) and thiourea (30 mmol) were dissolved in distilled water (35 mL) under vigorous stirring to form a homogeneous solution. After being stirred for 30 min, the solution was transferred into a 50 mL Teflon-lined stainless steel autoclave and maintained at 180 °C for 24 h.The obtained products were collected by centrifugation, washed with distilled water and ethanol, and dried at 40 °C under vacuum. The obtained MoS_2_ nanosheets (30 mg) was dispersed in ethyl alcohol (75 mL) and then iron acetylacetone (0.5 mmol), distilled water (1.8 mL) and ammonia (2 mL) were added. After sonication for 15 min at room temperature the mixture was heated at 80 °C for 10 h in a water bath. The precipitates were separated by centrifugation, washed with distilled water and ethanol, and dried at 40 °C for 24 h under vacuum. The growth of ultra-small Fe_2_O_3_ nanoparticles on the MoS_2_ nanosheets was achieved after the dried powder was thermally treated at 500 °C for 3 h under an Ar flow. For convenience, the obtained sample denoted as Fe_2_O_3_/MoS_2_ composite. As shown in Figure S13, the Fe and Mo atom ratio was 2:1. The Fe_2_O_3_ was prepared by heating graphene–hollow iron oxide at 500 °C for 1.5 h under air atmosphere^72^, and then was thermally treated at 350 °C for 1 h under an Ar/H_2_ flow. The physical mixture Fe_2_O_3_ and MoS_2_ (denoted as Fe_2_O_3_+MoS_2_) as reference sample was prepared by grinding the MoS_2_ nanosheets and commercial Fe_2_O_3_ powder according to Fe and Mo atomic ratio.

### Structure characterizations

The morphology and size of the samples were characterized by scanning electron microscope (SEM, Hitachi SU 70) (Condition = Vacc = 15KV, Mag = x60.0k- x250k, Working Distance = 15800 um, Emission Current = 28000 nA) and a FEI Tecnai-F20 transmission electron microscope (TEM) equipped with a Gatan imaging filter (GIF), operated at an accelerating voltage of 200 kV, combined with HRTEM and EDX measurements. The crystal structure of the sample was determined by X-ray diffraction (XRD) [D/max 2550 V, Cu Kα radiation] in the 2θ range of 5–70°. (X-Ray 40 kV/100 mA, DivSlit 1 deg., RecSlit open, DivH.L.Slit 10mm, SctSlit 8.0mm, Step 0.02). X-ray photoelectron spectra (XPS) were carried out by using a spectrometer with Mg Kα radiation (PHI 5700 ESCA System). The binding energy was calibrated with the C 1 s position of contaminant carbon in the vacuum chamber of the XPS instrument (284.6 eV). The pore diameter distribution and surface area were tested by nitrogen adsorption/desorption analysis (TRISTAR II3020).

### Electrochemical measurements

The electrochemical tests were performed at ambient temperature using two-electrode coin cells (CR 2016) with lithium foils serving as the counter electrode. The active material was mixed with a conductive acetylene black, and a commercial polymer binder (LA133) at a weight ratio of 70:15:15. The mixture was painted onto a Cu foil and dried in air, then cut into 14 mm diameters of round piece. Finally the electrode pieces were dried in vacuum at 60 °C for 12 h to adequately evaporate the residual moisture. The average thickness and mass loading of the electrode were ~3.5 *μ*m (Figure S14) and 1.5 ± 0.2 mg, respectively. The electrolyte was made of LiPF_6_ (1 M) in a mixture of ethylene carbonate (EC) and dimethyl carbonate (DMC) with the volume ratio 1:1. The 2016 coin-type cells were assembled in an Ar filled glove box, and pure Li foils were used as the counter electrodes. The charge–discharge cycles were carried out on a battery measurement system (LAND-BT2013A) at various current densities of 100–5000 mA g^−1^ in the cutoff voltage range of 3 to 0 V versus Li/Li^+^ at room temperature (~20 °C). Cyclic voltammetry measurements were carried out on a CHI660D electrochemical workstation over the potential range of 3.0 to 0.01 V at a scan rate of 1 mV s^−1^.

## Additional Information

**How to cite this article**: Qu, B. *et al*. Ultrasmall Fe_2_O_3_ nanoparticles/MoS_2_ nanosheets composite as high-performance anode material for lithium ion batteries. *Sci. Rep.* **7**, 42772; doi: 10.1038/srep42772 (2017).

**Publisher's note:** Springer Nature remains neutral with regard to jurisdictional claims in published maps and institutional affiliations.

## Supplementary Material

Supplementary Information
