# Supplementary material for: Ultrasmall Fe2O3 nanoparticles/MoS2 nanosheets composite as high-performance anode material for lithium ion batteries
Source: Sci Rep. 2017 Feb 20;7:42772. doi: 10.1038/srep42772 (PMC5316998; doi:10.1038/srep42772)
Supplement: Supplementary Information [file srep42772-s1.doc]

**Supplementary Information**

Ultrasmall Fe_2_O_3_ nanoparticles/MoS_2_ nanosheets composite as high-performance anode material for lithium ion batteries

Bin Qu,^1, 2^ Yue Sun,^1^ Lianlian Liu,^1^ Chunyan Li,^1,*^ Changjian Yu,^1^ Xitian Zhang^2^, & Yujin Chen^1,*^

^1^Key Laboratory of In-Fiber Integrated Optics, Ministry of Education and College of Science, Harbin Engineering University, Harbin 150001, China.

^2^ Key Laboratory for Photonic and Electronic Bandgap Materials, Ministry of Education and School of Physics and Electronic Engineering, Harbin Normal University, Harbin 150025, China.

*Correspondence

Yujin Chen, Key Laboratory of In-Fiber Integrated Optics, Ministry of Education and College of Science, Harbin Engineering University, Harbin 150001, China. E-mail: chenyujin@hrbeu.edu.cn and chunyanli@hrbeu.edu.cn / Tel: +86-451-82519754.

/ Fax: +86-451-82519754.

Chunyan Li, Key Laboratory of In-Fiber Integrated Optics, Ministry of Education and College of Science, Harbin Engineering University, Harbin 150001, China. E-mail: chunyanli@hrbeu.edu.cn.

**KEYWORDS**: molybdenum disulfide, iron oxide, anode, lithium-ion battery

**
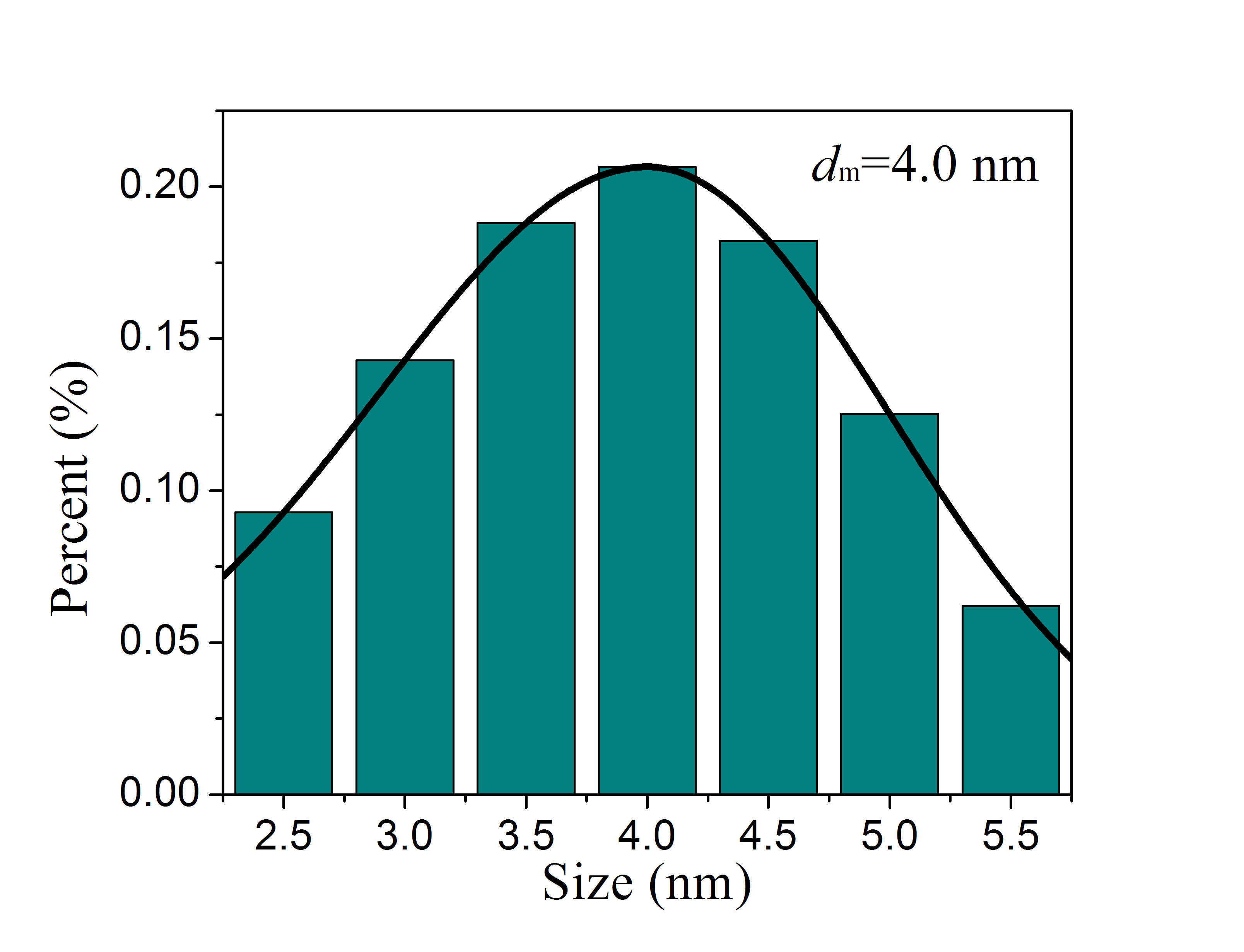
**

**Figure S1** The Fe_2_O_3_ particle size distribution.

**
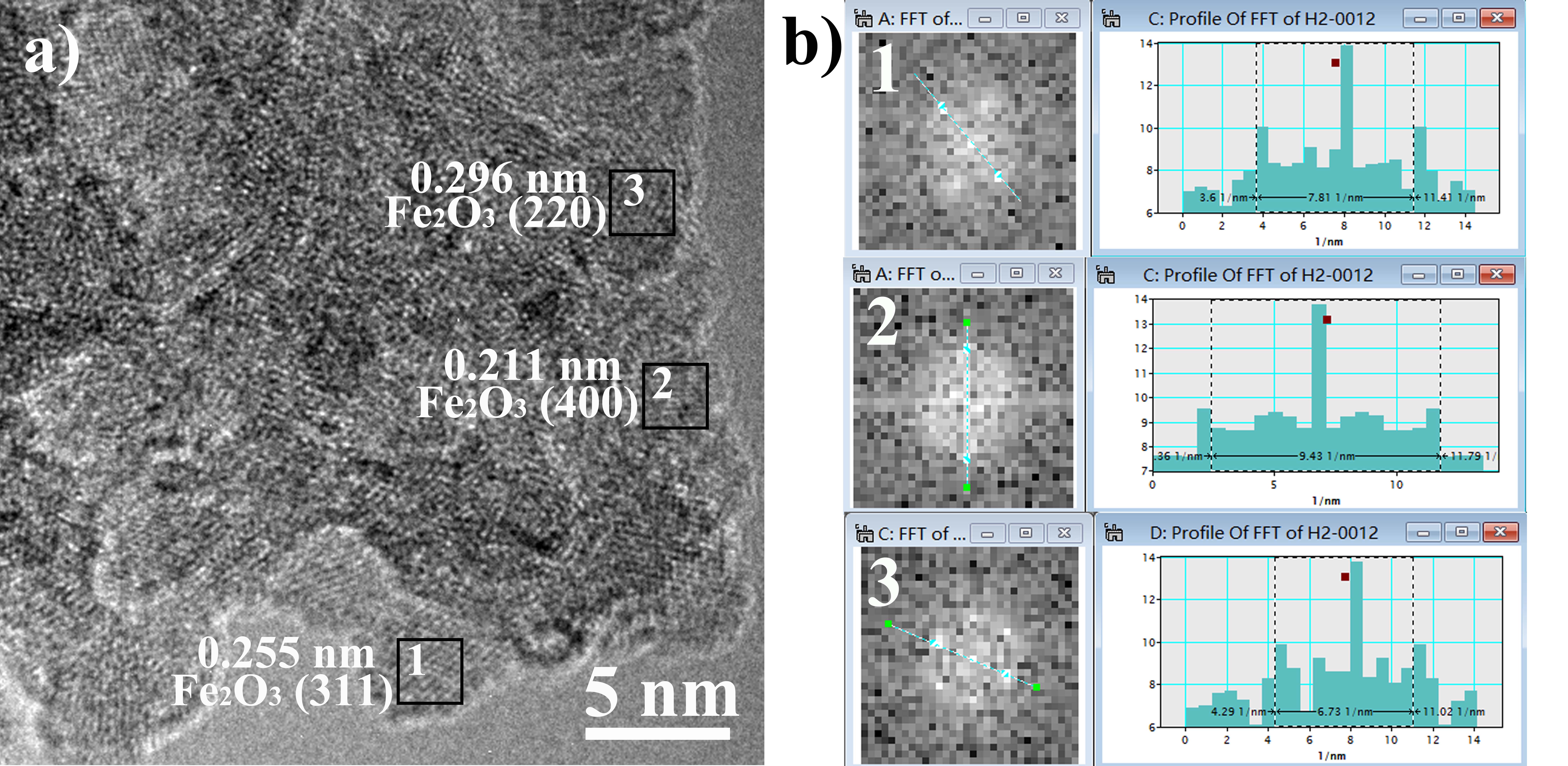
**

**Figure S2** a) HRTEM images taken from basal plane of the Fe_2_O_3_/MoS_2_ composite**,** b) fast Fourier transformation image of the Fe_2_O_3_/MoS_2_ composite**.**

**
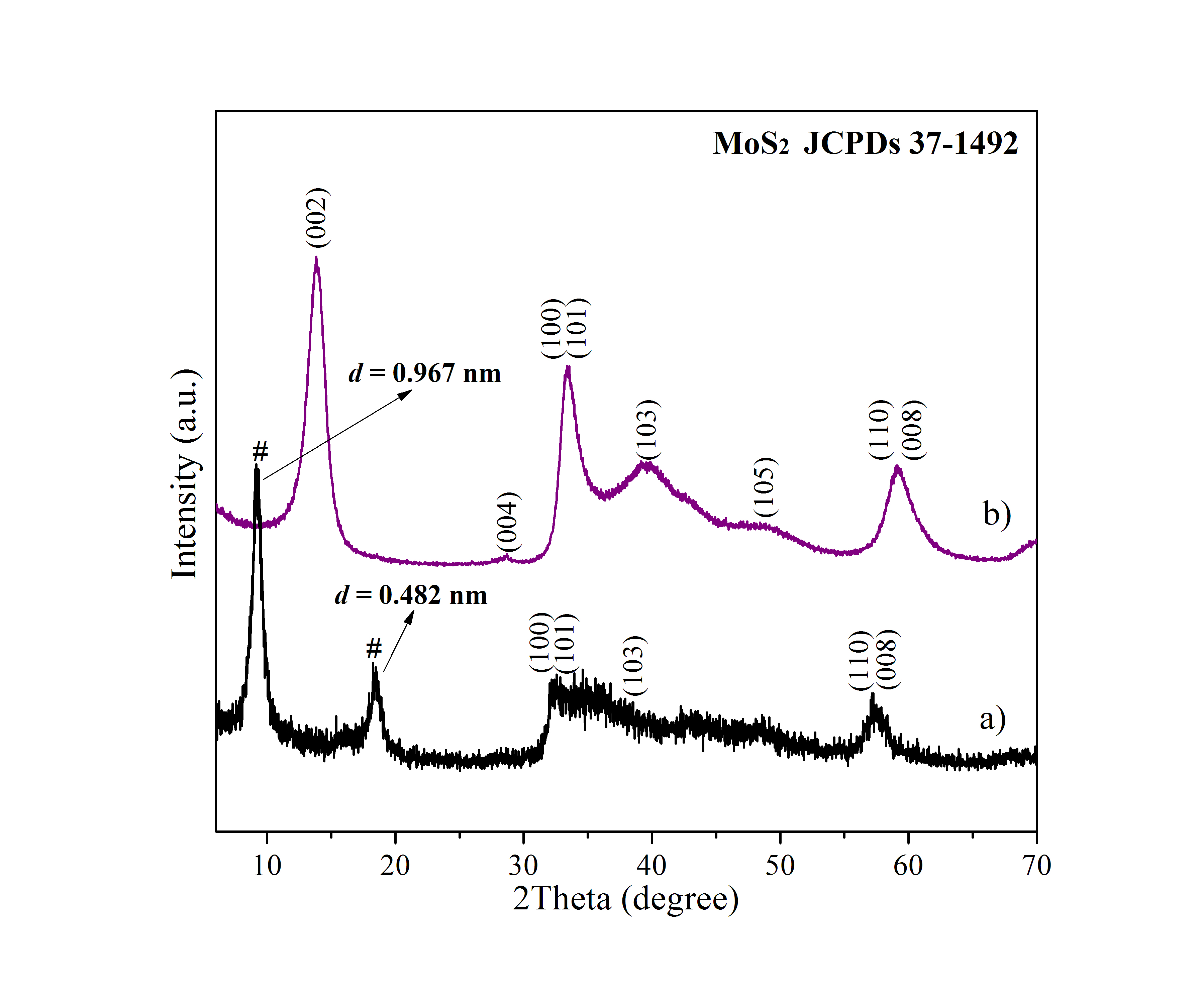
**

**Figure S3** XRD patterns. a) MoS_2_ nanosheets, b) MoS_2_ nanosheets annealed at 500°C for 3 h.

**
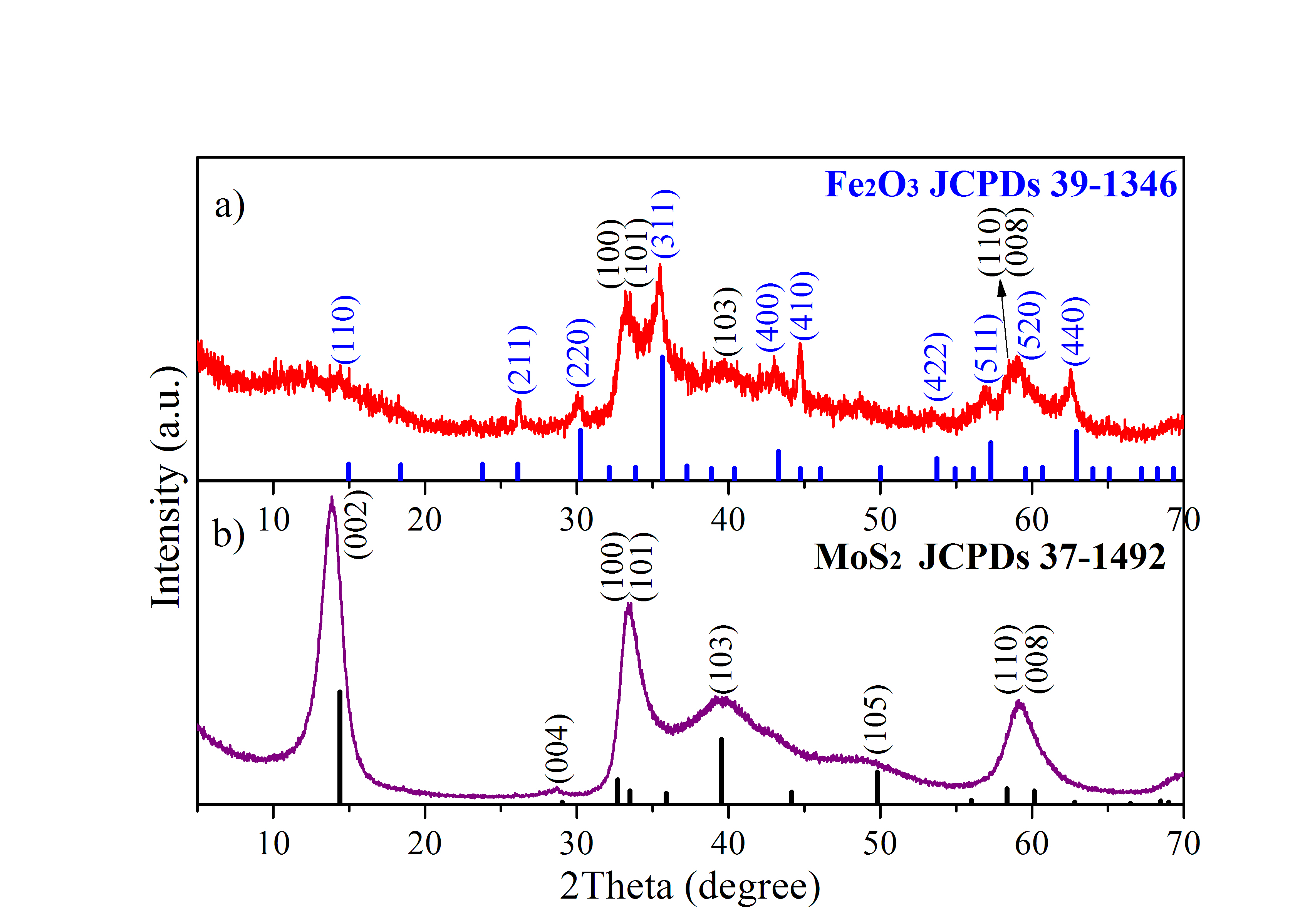
**

**Figure S4** XRD patterns. a) Fe_2_O_3_/MoS_2_ composite, b) MoS_2_ nanosheets annealed at 500°C for 3 h.

**
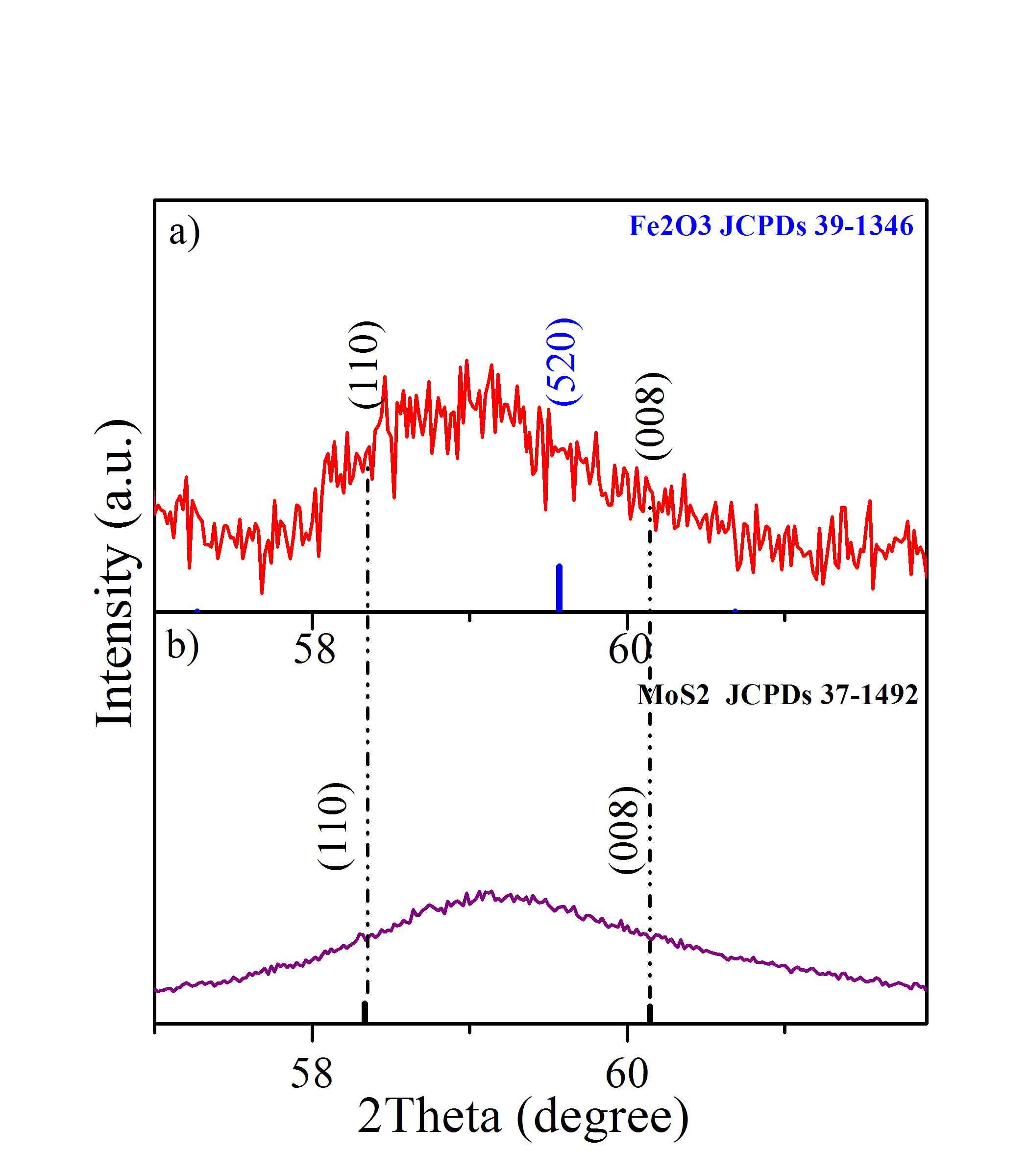
**

**Figure S5** XRD patterns. a) Fe_2_O_3_/MoS_2_ composite, b) MoS_2_ nanosheets annealed at 500°C for 3 h.

**
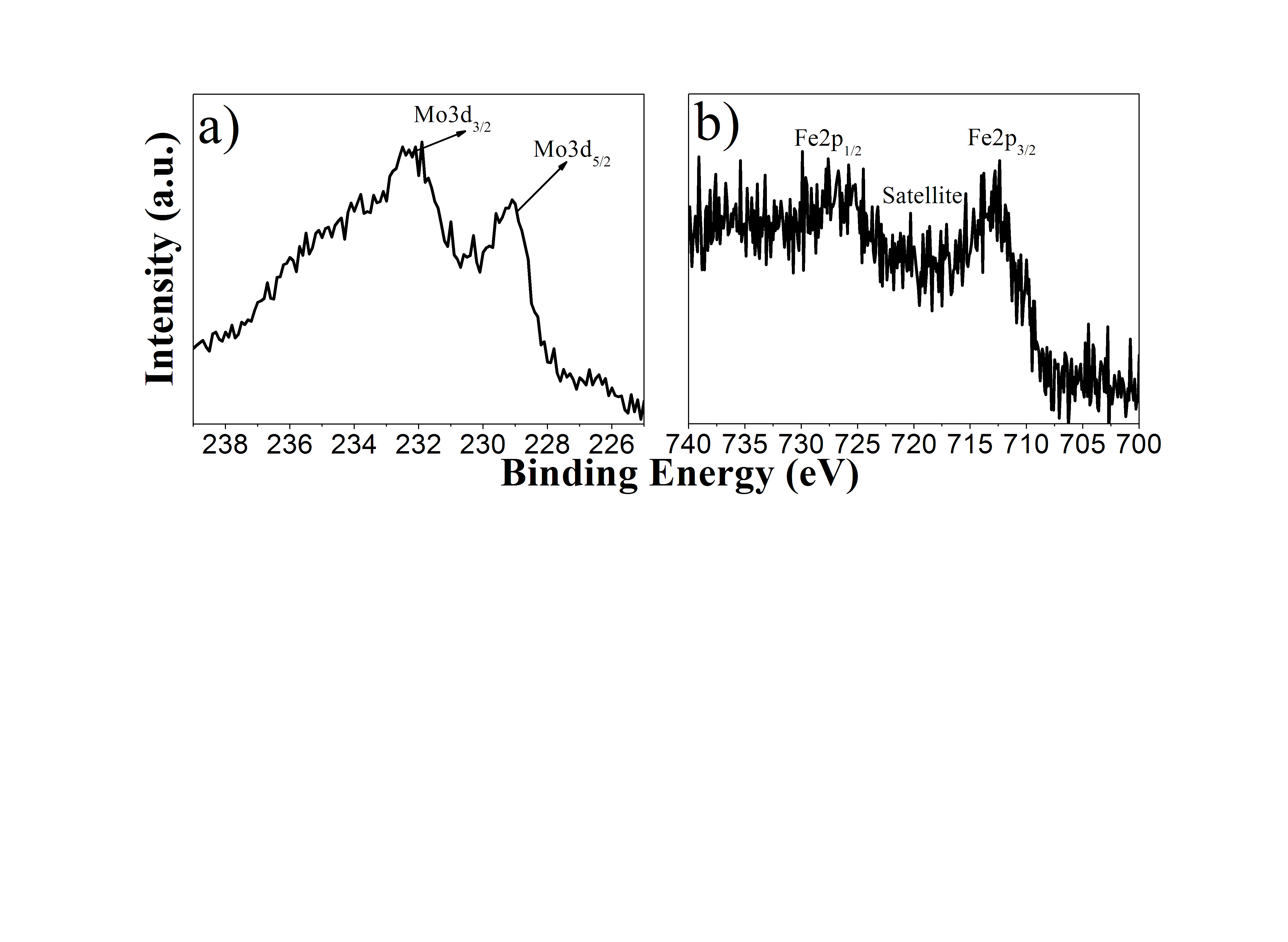
**

**Figure S6** XPS spectra of the physical mixture Fe_2_O_3_+MoS_2_ with the pristine nanosheets. a) Mo 3d XPS spectrum and b) Fe 2p XPS spectrum**.**

**
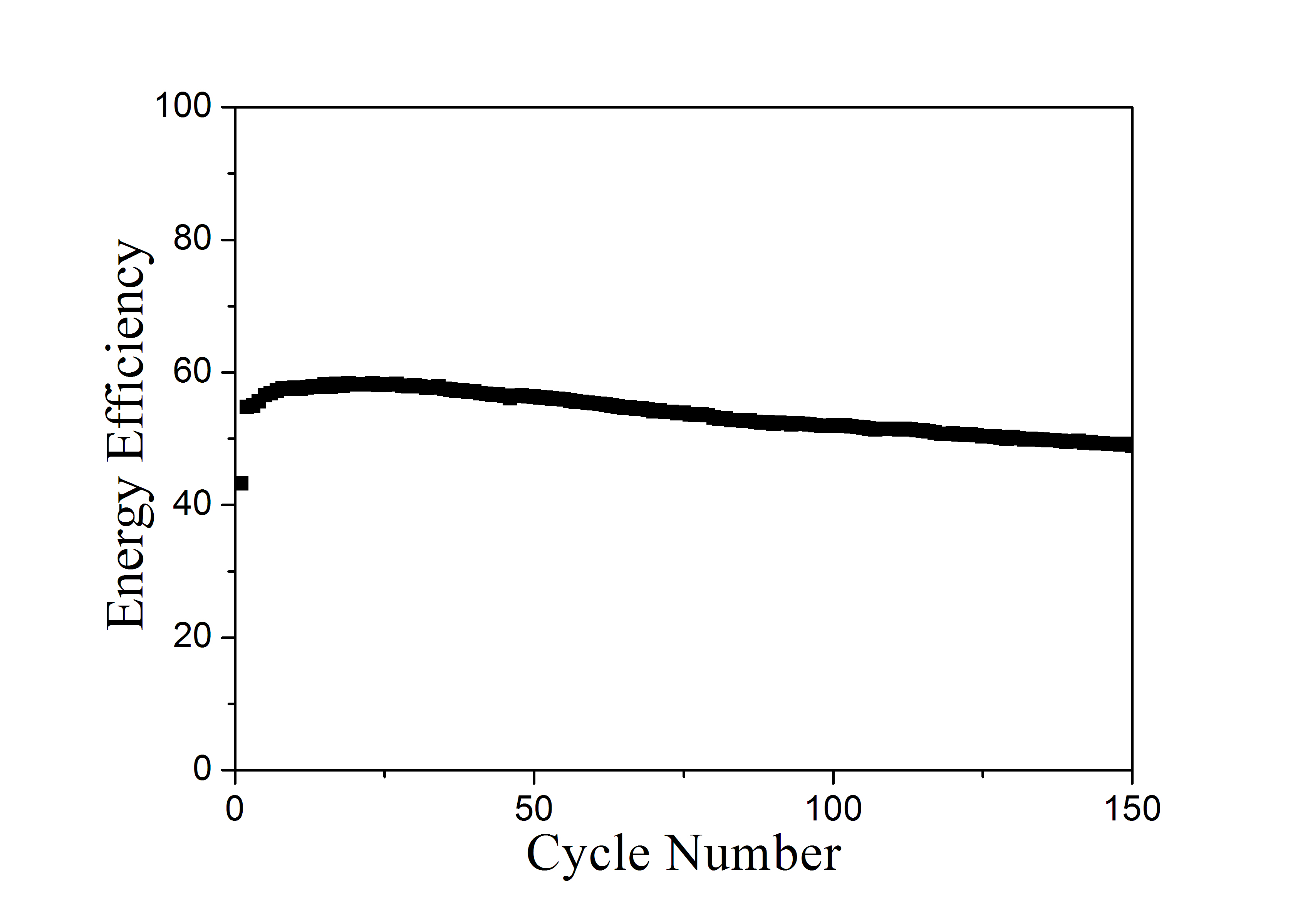
**

**Figure S7** The energy efficiency of the Fe_2_O_3_/MoS_2_ composite.

**
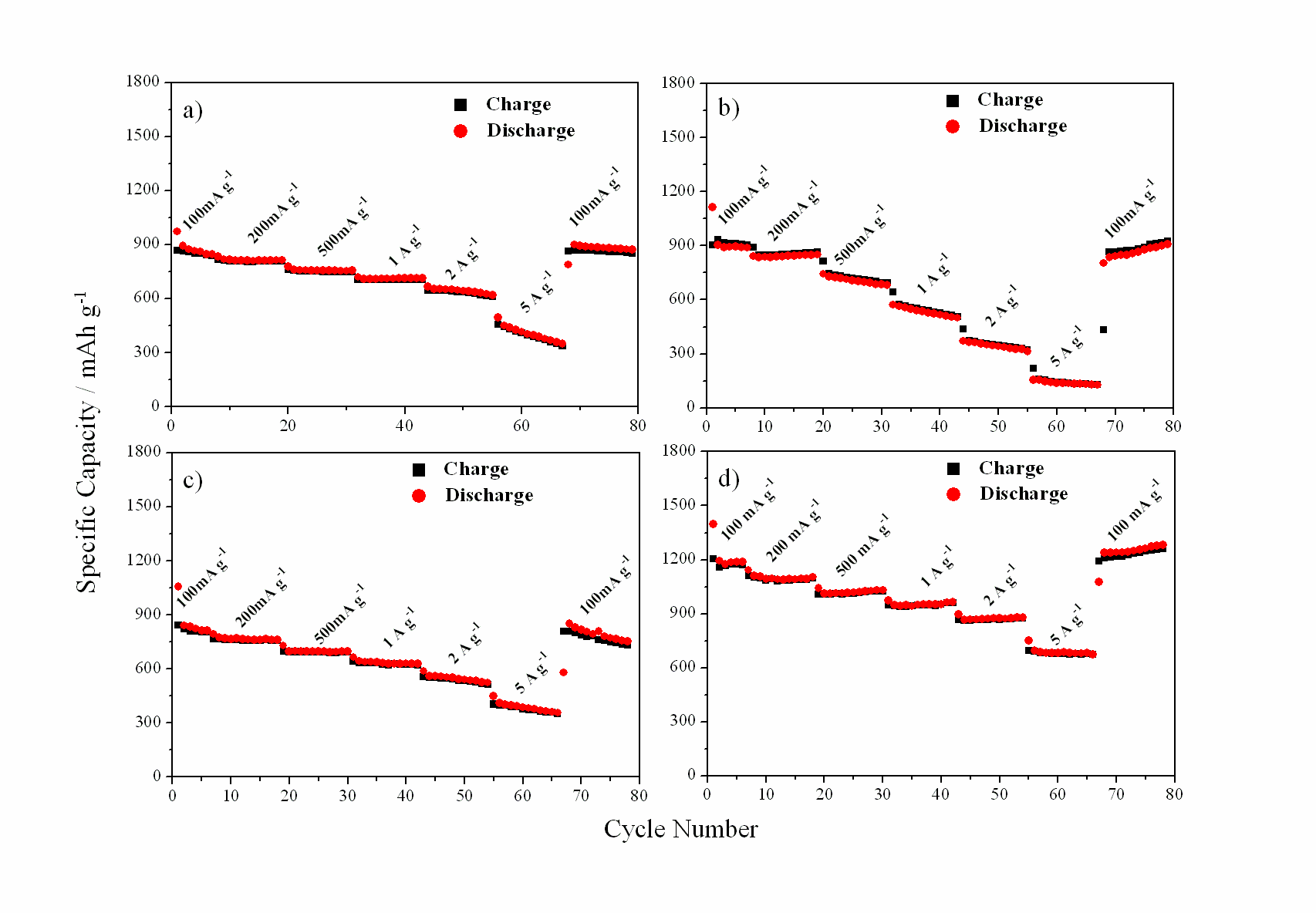
**

**Figure S8** Rate capabilities of samples. a) MoS_2_ nanosheets, b) Fe_2_O_3_, c) the physical mixture Fe_2_O_3_+MoS_2_ and d) Fe_2_O_3_/MoS_2_ composite.

**Table S1.** Comparison of capacity and rate capability of our prepared MoS_2_/Fe_2_O_3_composite with previously published LIB anode materials.

| Materials | Current density (mA g^-1^) | Cycle number | Reversible capacity (mAh g^−1^ ) | Refs. |
| --- | --- | --- | --- | --- |
| hierarchical MoS_2_ | 100 | 50 | 900 | Ref. 8 |
| MoS_2_ microboxes | 100 | 50 | 900 | Ref. 13 |
| MoS_2_/Graphene | 100 | 100 | 1020 | Ref. 21 |
| Fe_2_O_3_ nanoparticles | 100 | 100 | 300 | Ref. 37 |
| 3D MoS_2_@Fe_3_O_4_ | 100 | 100 | 1113 | Ref. 61 |
| Ag/Fe_3_O_4_–MoS_2_ | 200 | 100 | 1233 | Ref. 63 |
| MoS_2_Microspheres | 100 | 100 | 690 | Ref. 64 |
| Fe_2_O_3_/Graphene | 100 | 100 | 1062 | Ref. 65 |
| Fe_2_O_3_ Microboxes | 200 | 30 | 945 | Ref. 66 |
| Ag–MoS_2_ | 100 | 50 | 930 | Ref. 67 |
| MoS_2_ nanosheet@TiO_2_ | 100 | 100 | 578 | Ref. 68 |
| CNTs–MoS_2_ | 100 | 30 | 737 | Ref. 69 |
| MoS_2_/SiO_2_/graphene | 100 | 100 | 1060 | Ref. 70 |
| MoS_2_/polyaniline | 100 | 100 | ~800 | Ref. 71 |
| Fe_2_O_3_/MoS_2_ composite | 100  1000 | 150  140 | 1350  1011 | This work |
|  | 2000 | 140 | 864 |  |

**
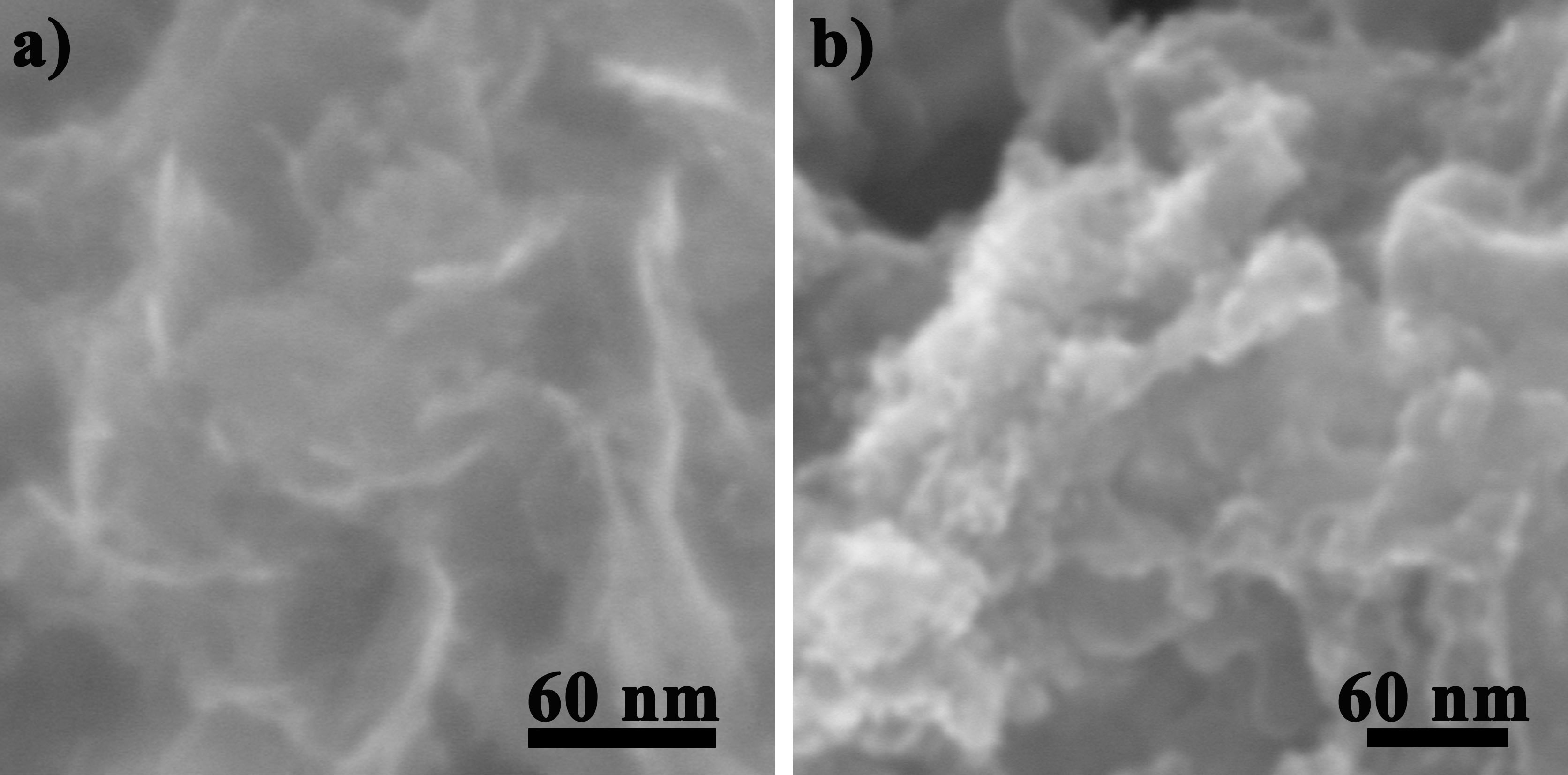
**

**Figure S9** The SEM images of the Fe_2_O_3_/MoS_2_ composite after 100 cycles at 1 A g^-1^. a) SEM image of the composite after fully charged process and b) SEM image of the composite after fully discharged process.


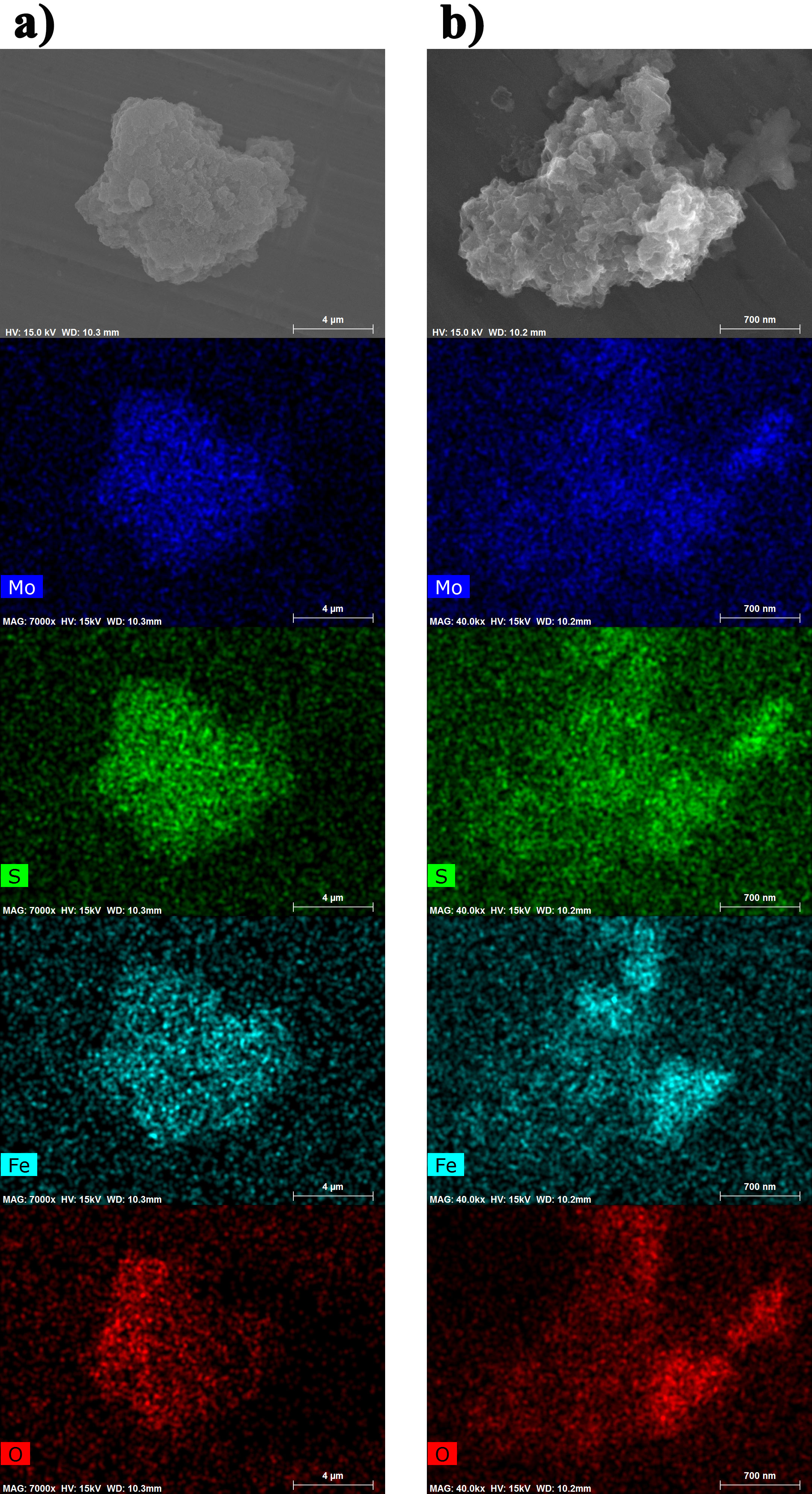


**Figure S10** The element mapping images of the Fe_2_O_3_/MoS_2_ composite after fully discharged process a) and after fully charged process b).

**
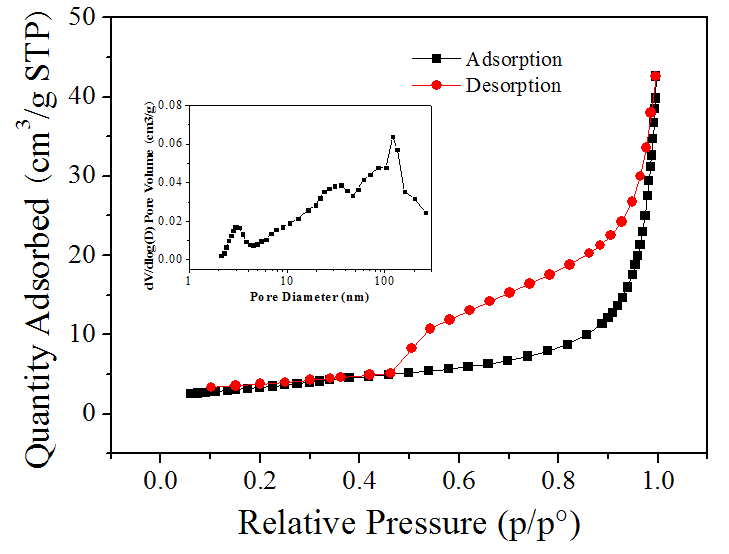
**

**Figure S11** Nitrogen adsorption/desorption isotherms of the pristine MoS_2_ nanosheets, and the inset shows the pore size distribution.

**
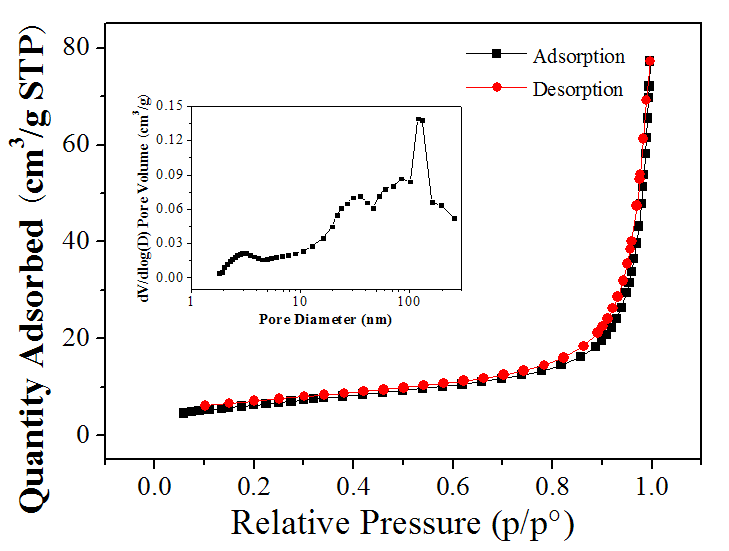
**

**Figure S12** Nitrogen adsorption/desorption isotherms of the Fe_2_O_3_/MoS_2_ composite, and the inset shows the pore size distribution.

| **element** | **Series** | **weight (%)** | **Atom (%)** |
| --- | --- | --- | --- |
| Fe | K | 32.44 | 20.43 |
| Mo | L | 27.40 | 10.04 |
| O | K | 23.13 | 50.85 |
| S | K | 17.03 | 18.68 |

**Figure S13** The EDS pattern of the Fe_2_O_3_/MoS_2_ composite**.**

**
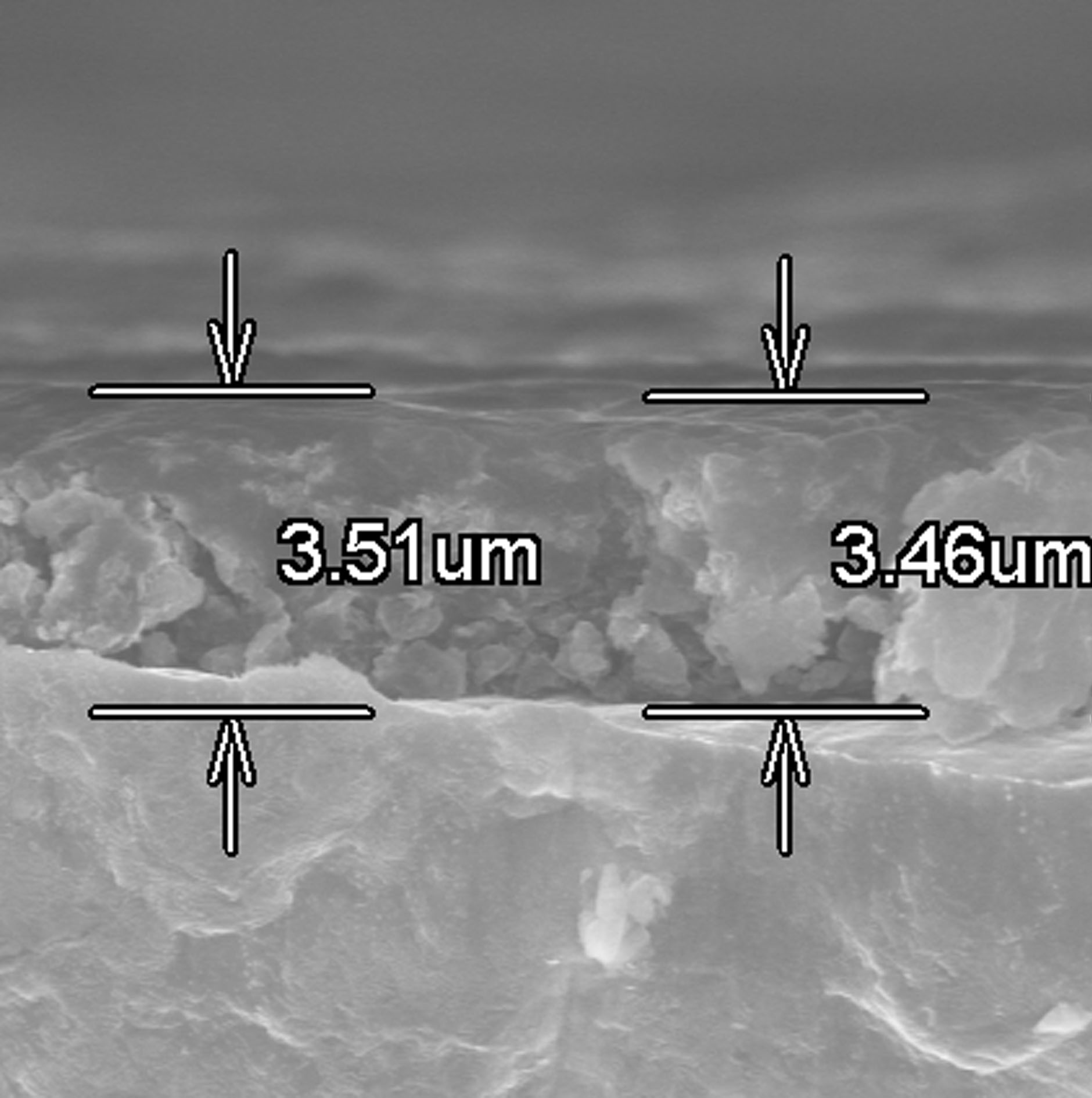
**

**Figure S14** Cross-section SEM images of the electrode pieces of the Fe_2_O_3_/MoS_2_ composite**.**
